# Supplementary material for: Unmasking AlphaFold to integrate experiments and predictions in multimeric complexes
Source: Nat Commun. 2024 Oct 9;15:8724. doi: 10.1038/s41467-024-52951-w (PMC11461844; doi:10.1038/s41467-024-52951-w)
Supplement: Supplementary file 3 — Description of Additional Supplementary Files [file 41467_2024_52951_MOESM3_ESM.pdf]

Description of Additional Supplementary Files

File Name: Supplementary Movie 1

Description: Video of partial opening of NF1. Interpolation between AF_unmasked models showing different positions of the GRD and Sec14-PH regions (shown as surfaces) relative to the helical platform (shown as cartoon). The two chains of NF1 are coloured in grey and purple.

File Name: Supplementary Movie 2

Description: Transition from the closed to the open position of NF1. Interpolation between AF_unmasked models from the closed to the open positions of the GRD and Sec14-PH regions, shown in gold and green respectively. Only one monomer is displayed.

File Name: Supplementary Movie 3

Description: 3D variability analysis of NF1 cryo-EM data, showing bending of the NF1 helical platform as well as appearance and disappearance of the GRD and Sec14-PH regions.
