# Supplementary material for: Unmasking AlphaFold to integrate experiments and predictions in multimeric complexes
Source: Nat Commun. 2024 Oct 9;15:8724. doi: 10.1038/s41467-024-52951-w (PMC11461844; doi:10.1038/s41467-024-52951-w)
Supplement: Supplementary file 7 — Reporting Summary [file 41467_2024_52951_MOESM7_ESM.pdf]

nature portfolio

Corresponding author(s): Claudio Mirabello

Last updated by author(s): Aug 26, 2024

Reporting Summary

Nature Portfolio wishes to improve the reproducibility of the work that we publish. This form provides structure for consistency and transparency in reporting. For further information on Nature Portfolio policies, see our Editorial Policies and the Editorial Policy Checklist.

Statistics

For all statistical analyses, confirm that the following items are present in the figure legend, table legend, main text, or Methods section.

n/a	Confirmed	
☐	☒	The exact sample size (*n*) for each experimental group/condition, given as a discrete number and unit of measurement
☐	☒	A statement on whether measurements were taken from distinct samples or whether the same sample was measured repeatedly
☒	☐	The statistical test(s) used AND whether they are one- or two-sided<br>*Only common tests should be described solely by name; describe more complex techniques in the Methods section.*
☐	☒	A description of all covariates tested
☒	☐	A description of any assumptions or corrections, such as tests of normality and adjustment for multiple comparisons
☐	☒	A full description of the statistical parameters including central tendency (e.g. means) or other basic estimates (e.g. regression coefficient) AND variation (e.g. standard deviation) or associated estimates of uncertainty (e.g. confidence intervals)
☒	☐	For null hypothesis testing, the test statistic (e.g. *F*, *t*, *r*) with confidence intervals, effect sizes, degrees of freedom and *P* value noted<br>*Give P values as exact values whenever suitable.*
☒	☐	For Bayesian analysis, information on the choice of priors and Markov chain Monte Carlo settings
☒	☐	For hierarchical and complex designs, identification of the appropriate level for tests and full reporting of outcomes
☒	☐	Estimates of effect sizes (e.g. Cohen's *d*, Pearson's *r*), indicating how they were calculated

Our web collection on statistics for biologists contains articles on many of the points above.

Software and code

Policy information about availability of computer code

Data collection	AlphaFold-Multimer (parameters versions v2.2 and v2.3) was used to perform baseline predictions. PDB benchmark data have been collected from the Protein Data Bank on January 19, 2024.
Data analysis	DockQ and iRMSD scores were obtained with DockQ v2.0. RMSD values were obtained with the TMscore tool (Fortran version). Q-scores were derived with UCFS ChimeraX v1.7

For manuscripts utilizing custom algorithms or software that are central to the research but not yet described in published literature, software must be made available to editors and reviewers. We strongly encourage code deposition in a community repository (e.g. GitHub). See the Nature Portfolio guidelines for submitting code & software for further information.

Data

Policy information about availability of data

All manuscripts must include a data availability statement. This statement should provide the following information, where applicable:

- Accession codes, unique identifiers, or web links for publicly available datasets
- A description of any restrictions on data availability
- For clinical datasets or third party data, please ensure that the statement adheres to our policy

The source data underlying Figures 2, 4c, 5c-d and Supplementary Figures S1-S16 are provided as a Source Data file.
All the predicted structures, along with log files, inputs and templates are available with DOI: 10.17044/scilifelab.24198669. ChimeraX sessions for the analyses of

Cryo-EM test cases are available with DOI: 10.17044/scilifelab.25653297.
NF1 videos referenced in text are available as Supplementary data files.
Deposited PDB structures referenced in the text are:
7ALW (https://doi.org/10.2210/pdb7ALW/pdb), 7QIJ (https://doi.org/10.2210/pdb7QIJ/pdb), 4K2H (https://doi.org/10.2210/pdb4K2H/pdb), 5IU0 (https://doi.org/10.2210/pdb5IU0/pdb), 1QVR (https://doi.org/10.2210/pdb1QVR/pdb), 5OG1 (https://doi.org/10.2210/pdb5OG1/pdb), 6RN3 (https://doi.org/10.2210/pdb6RN3/pdb), 7PGU (https://doi.org/10.2210/pdb7PGU/pdb), 6OB3 (https://doi.org/10.2210/pdb6OB3/pdb), 1NF1 (https://doi.org/10.2210/pdb1NF1/pdb), 3PEG (https://doi.org/10.2210/pdb3PEG/pdb).

Research involving human participants, their data, or biological material

Policy information about studies with human participants or human data. See also policy information about sex, gender (identity/presentation), and sexual orientation and race, ethnicity and racism.

Reporting on sex and gender	*Use the terms sex (biological attribute) and gender (shaped by social and cultural circumstances) carefully in order to avoid confusing both terms. Indicate if findings apply to only one sex or gender; describe whether sex and gender were considered in study design; whether sex and/or gender was determined based on self-reporting or assigned and methods used. Provide in the source data disaggregated sex and gender data, where this information has been collected, and if consent has been obtained for sharing of individual-level data; provide overall numbers in this Reporting Summary. Please state if this information has not been collected. Report sex- and gender-based analyses where performed, justify reasons for lack of sex- and gender-based analysis.*
Reporting on race, ethnicity, or other socially relevant groupings	*Please specify the socially constructed or socially relevant categorization variable(s) used in your manuscript and explain why they were used. Please note that such variables should not be used as proxies for other socially constructed/relevant variables (for example, race or ethnicity should not be used as a proxy for socioeconomic status). Provide clear definitions of the relevant terms used, how they were provided (by the participants/respondents, the researchers, or third parties), and the method(s) used to classify people into the different categories (e.g. self-report, census or administrative data, social media data, etc.) Please provide details about how you controlled for confounding variables in your analyses.*
Population characteristics	*Describe the covariate-relevant population characteristics of the human research participants (e.g. age, genotypic information, past and current diagnosis and treatment categories). If you filled out the behavioural & social sciences study design questions and have nothing to add here, write "See above."*
Recruitment	*Describe how participants were recruited. Outline any potential self-selection bias or other biases that may be present and how these are likely to impact results.*
Ethics oversight	*Identify the organization(s) that approved the study protocol.*

Note that full information on the approval of the study protocol must also be provided in the manuscript.

Field-specific reporting

Please select the one below that is the best fit for your research. If you are not sure, read the appropriate sections before making your selection.

☒ Life sciences ☐ Behavioural & social sciences ☐ Ecological, evolutionary & environmental sciences

For a reference copy of the document with all sections, see nature.com/documents/nr-reporting-summary-flat.pdf

Life sciences study design

All studies must disclose on these points even when the disclosure is negative.

Sample size	In the benchmarking, sample sizes are the number of unique protein dimers for which predictions were performed. Only dimers that were not included in the training of AlphaFold-Multimer (v2.3) could be included. To avoid biases, the set of dimers was redundancy-reduced through structural comparisons (see below) to ensure sufficient diversity.
Data exclusions	Out of 633 heterodimers initially in the PDB benchmark, 382 were excluded after a structural redundancy reduction procedure (described in the Methods). When multiple heterodimers were similar, only one representative was kept. Of the final 251 heterodimers in the first dataset, 28 were kept in the final benchmark of PDB homologs, as they were the only ones were the regular version of AlphaFold-Multimer could not produce a good prediction (see Methods).
Replication	For all predictions in the benchmarking, each structure was predicted five times for each of the five neural network models, for a total of 25 predictions. Only one prediction with top predicted ranking confidence was evaluated.
Randomization	Samples in this study are not allocated into experimental groups, as all samples are tested (predicted) in all conditions. Randomization was not necessary.
Blinding	See above, all samples are tested in all conditions and blinding was not necessary.

Reporting for specific materials, systems and methods

We require information from authors about some types of materials, experimental systems and methods used in many studies. Here, indicate whether each material, system or method listed is relevant to your study. If you are not sure if a list item applies to your research, read the appropriate section before selecting a response.

Materials & experimental systems

n/a	Involved in the study
☒	☐ Antibodies
☒	☐ Eukaryotic cell lines
☒	☐ Palaeontology and archaeology
☒	☐ Animals and other organisms
☒	☐ Clinical data
☒	☐ Dual use research of concern
☒	☐ Plants

Methods

n/a	Involved in the study
☒	☐ ChIP-seq
☒	☐ Flow cytometry
☒	☐ MRI-based neuroimaging

Plants

Seed stocks	*Report on the source of all seed stocks or other plant material used. If applicable, state the seed stock centre and catalogue number. If plant specimens were collected from the field, describe the collection location, date and sampling procedures.*
Novel plant genotypes	*Describe the methods by which all novel plant genotypes were produced. This includes those generated by transgenic approaches, gene editing, chemical/radiation-based mutagenesis and hybridization. For transgenic lines, describe the transformation method, the number of independent lines analyzed and the generation upon which experiments were performed. For gene-edited lines, describe the editor used, the endogenous sequence targeted for editing, the targeting guide RNA sequence (if applicable) and how the editor was applied.*
Authentication	*Describe any authentication procedures for each seed stock used or novel genotype generated. Describe any experiments used to assess the effect of a mutation and, where applicable, how potential secondary effects (e.g. second site T-DNA insertions, mosiacism, off-target gene editing) were examined.*
